# Approach to the older patient with cancer

**DOI:** 10.1186/1741-7015-11-218

**Published:** 2013-10-10

**Authors:** Maxine de la Cruz, Eduardo Bruera

**Affiliations:** 1 Department of Palliative Care and Rehabilitation Medicine, The University of Texas MD Anderson Cancer Center, 1515 Holcombe Boulevard Unit 1414, Houston, TX 77030, USA

**Keywords:** Advance cancer, Geriatric patient, Palliative care, Supportive care, Symptom management

## Abstract

The incidence of cancer increases with advanced age. And as the world population ages, clinicians will be faced with a growing number of older patients with cancer. The challenge that clinicians face involves carefully choosing the type of therapeutic care plan that is most appropriate given a person’s level of physical reserve, medical comorbidities, and psychosocial resources. Inclusion of assessment tools in clinical practice such as a comprehensive geriatric assessment can assist clinicians in identifying patients who will benefit from aggressive cancer care or palliative measures. The role of palliative care, especially in the frail older patient, is critical in improving quality of life. Improvement in best care practices in older patients with cancer requires their inclusion in clinical trials.

## Background

The incidence of cancer increases dramatically with age. Current statistics show that about 50% of cancers diagnosed in the United States occur in people over the age of 65 and this is expected to rise to 70% by 2030. The incidence of cancer is reported to be 12 to 36 times higher in patients 65 years or older compared with those aged 25 to 44 years, and 2 to 3 times higher in individuals 45 to 64 years of age. Mortality is also higher in older patients, accounting for nearly 70% of cancer deaths per year [1]. The challenge for cancer specialists is to determine the optimum treatment for elderly patients, a heterogeneous population in terms of comorbidity, disability, physical reserve, and other geriatric conditions. Best treatment practices for older patients with cancer are lacking, in part due to sub-optimal or excessively toxic treatments and under-representation in clinical trials, resulting in poorer outcomes compared with younger patients. Issues that impact oncologic and supportive care management unique to the geriatric population will be highlighted in this article.

## Discussion

Aging is defined as a progressive loss of a person’s entropy and fractal organization that limits the functional reserve of multiple organ systems and the ability to withstand stress. Such changes bring about reduced life expectancy, increased susceptibility to disease and loss of personal independence. The process of aging varies from person to person. Some patients undergo successful aging, where robust physical and cognitive abilities are maintained throughout life [2]. There are those described as having frailty, which is characterized by weakness and decreased functional reserve [3,4]. Physiologic age does not correspond to a person’s chronologic age and is more clinically useful in medical decision-making. Determination of physiologic age is largely based on a comprehensive geriatric assessment (CGA). This multi-dimensional method incorporates functional status, comorbidities, cognition, nutrition, medications, and social support structures. It assists clinicians to better estimate life expectancy and functional reserve, which are relevant in treatment-related decisions. The use of the CGA also allows for identification of frailty and other unrecognized conditions that can influence management.

The older patient diagnosed with cancer can present with various other problems, making evaluation of treatment options for cancer more complex. Besides consideration of cancer-related factors, assessment of other aspects of a patient’s health status including social, functional, cognitive, economic and spiritual domains, is crucial in developing a treatment plan that is tailored to individual needs. Since the mid-1990s, oncologists and geriatricians have sought to integrate the use of CGA in the oncologic setting, hoping to assist clinicians in formulating optimal therapeutic plans. Components of the CGA have been shown to not only affect life expectancy and function but also tolerance to treatment and symptom burden. Box 1 lists questions that can affect therapeutic choices and measure treatment efficacy and guide in planning care for the elderly [5].

### Box 1 Questions to ask in deciding therapeutic care plan for the elderly cancer patient [6]

Is the patient going to die of cancer or with cancer?

Is the patient going to live long enough to suffer the consequences of cancer?

Is the patient able to tolerate the treatment?

What are the long term consequences of cancer treatment in the elderly?

Will any treatment improve the quality of life?

What are the patient’s goals of care?

Is the social network of the patient adequate to support him/her during the treatment?

Frailty is a concept that is becoming increasingly used to describe patients with reduced reserve in multiple organ systems resulting in increased risk of disability or death with minor stresses. Functionally, these patients are characterized as having a wasting syndrome; low energy, gait speed and grip strength; many chronic medical comorbidities; complex psychosocial problems; significant risk of dependency; and other adverse health outcomes [7-9]. Cancer and its treatment are substantial stressors that diminish physiological reserves, so the concept of frailty is particularly pertinent for older patients with cancer. On the basis of the frailty phenotype, optimal management strategies for treatment and inclusion in clinical trials may be achieved through patient stratification. Table 1 shows Fried’s criteria for frailty [8]. Fit geriatric patients are good candidates for almost the same type of cancer treatment as younger patients and have similar survival outcomes. Patients who are frail would benefit from more palliative approaches to treatment [8], with the assumption that their functional reserve is so poor that they will not be able to tolerate even minimal stress that may arise from therapeutic regimens. Those who fall in between are more problematic and would require individualization of treatment approach.

**Table 1:** **Fried’s frailty criteria**^1 ^[7]

**Abnormalities**	**Frailty scale**
Involuntary weight loss of 10 lbs or more in the last 6 months	Fit (no abnormalities)
Reduced grip strength	Pre-frail (2 abnormalities or less)
Difficulty initiating movements	Frail (3 or more abnormalities)
Reduced walking speed	
Fatigue	

1. Categories of Frailty:

Fit: No abnormalities.

Pre-Frail: 2 abnormalities or less.

Frail: 3 or more abnormalities.

Frailty has rarely been measured in patients with cancer. Some evidence shows that frailty is present in younger patients with cancer, and can even be observed in cancer survivors. This indicates that familiarity and understanding of the concept of frailty is essential from the time of consideration of treatment to cure and subsequent follow-ups. Most older adults with cancer will fall into the pre-frail and frail category, therefore it is important that supportive care measures be made available to them. This requires access to palliative care services to manage symptoms that may arise from the burden of disease as well as the adverse effects of cancer treatment. The interdisciplinary team that is central to the practice of palliative care is most useful in the geriatric patient. These patients will benefit from specialized care encompassing multiple domains for improved quality of life. Quantifying frailty can help reduce futile and burdensome interventions that are not expected to improve symptoms or those that may worsen function and quality of life. Frailty assessment is therefore essential for the timely delivery of holistic palliative care in geriatric patients with progressive and terminal disease [10].

With the aging of the population and the known association between cancer and older age, inclusion of older geriatric patients in clinical trials has been advocated by several experts. The under-representation of this at-risk population has been highlighted by several authors [11]. It has been proposed that exclusion in studies is related to reduced function, presence of comorbidities, concomitant medications, lack of access, and poor social support. Proposals to improve participation of older adults include clinical trials specifically designed for older adults with multiple comorbidities, trials with dosing regimens tailored for older patients, standardized use of geriatric tools, and alternative end points or outcomes [12-14]. Data from 2001 to 2010 showed that 24 drugs were approved by the US Food and Drug Administration for cancer treatment and only 33% of patients that were registered in those trials were 65 years or older [15]. This is a dismal representation given that 59% of patients with cancer during the same time period were of this age group [16]. The problem with non-inclusion is that evidence-based treatment recommendations are not always applicable to the older patient, particularly those who are vulnerable and frail. In addition, those included in the trials are not representative of the majority of older adults with cancer. Designing protocols tailored to include older patients should be specially designed to avoid under-representation. The CGA can be used as a tool that can classify patients fit to receive certain specific treatments other than using age and clinical judgment as the sole basis for treatment decisions. Such clinical trials would be helpful in guiding appropriate patient selection, risk stratification, and toxicity evaluation.

## Conclusion

The incidence of cancer rises with increasing age. Specialists who care for cancer patients face the difficulty in identifying optimum treatment options for elderly patients. The concept of frailty and the use of the CGA help guide treatment decisions, enabling clinicians to better identify the presence of comorbidities and geriatric syndromes, and the level of physical reserve or disability. Incorporating supportive care measures early in these patients, particularly those who fall into the pre-frail and frail categories of the frailty index, is crucial to successful management. Future cancer and palliative care research should be more inclusive of this population. Specially designed protocols for older adults are necessary to provide more appropriate evidence-based approaches to cancer treatment in geriatrics.

## Abbreviations

CGA: Comprehensive geriatric assessment.

## Competing interests

MC does not have any competing interests. EB is supported in part by National Institutes of Health grants (RO1NR010162-01A1, RO1CA122292-01, RO1CA124481-01) and in part by the MD Anderson Cancer Center support grant #CA 016672.

## Authors’ contributions

Both authors were involved in the conceptualization of the manuscript, its writing and subsequent editing. Both authors read and approved the final manuscript.

## Pre-publication history

The pre-publication history for this paper can be accessed here:

http://www.biomedcentral.com/1741-7015/11/218/prepub
